# A systematic review of menopause apps with an emphasis on osteoporosis

**DOI:** 10.1186/s12905-023-02612-9

**Published:** 2023-09-29

**Authors:** Deborah Paripoorani, Norina Gasteiger, Helen Hawley-Hague, Dawn Dowding

**Affiliations:** 1 EMERGING Research Team, Manchester Royal Infirmary, Manchester, UK; 2 National Institute for Health and Care Research, Applied Research Collaboration Greater Manchester, School of Health Sciences, Faculty of Biology, Medicine and Health, The University of Manchester, Manchester, UK; 3 Division of Nursing, Midwifery and Social Work, School of Health Sciences, The University of Manchester, Manchester, UK; 4 Division of Informatics, Imaging & Data Sciences, School of Health Sciences, The University of Manchester, Manchester, UK

**Keywords:** Menopause, Osteoporosis, Smartphone, Mobile health, Review, Menopause app, Women’s health

## Abstract

**Background:**

Menopause can significantly hasten bone loss. Mobile phones provide an efficient way to manage, track and understand menopause using apps. A previous review of menopause apps found numerous apps designed to help women manage menopause. However, it did not use validated measures to assess the quality of the apps and did not focus on content related to osteoporosis.

**Methods:**

This app review aligns with the updated Preferred Reporting Items for Systematic Reviews and Meta-Analysis guidelines. The keywords used to search for the apps were “menopause” and “menopausal”. Apps were included if they were in English, for individuals or groups and had a lifestyle focus. Apps that looked at other aspects of women’s health, required external devices, cost to download, or were symptom-tracking were excluded. The quality and functionality were assessed using the Mobile App Rating Scale and IMS Institute for Healthcare Informatics Functionality score. Data were synthesised descriptively.

**Results:**

Twenty-eight apps were selected and reviewed from the 236 apps screened from the Apple store and Google play store. Only 57% of the apps reviewed (*n* = 16) had content on osteoporosis which was educational in purpose. The readability of the apps was complex and best understood by university graduates. The average functionality score of the apps reviewed was 4.57 out of 11 and that of quality is 3.1 out of 5, both of which need improvement.

**Conclusions:**

Existing menopause apps need more input from experts to improve the quality and functionality, using simple language. More emphasis on specific health problems during menopause, including osteoporosis, is required.

**Trial registration:**

Not relevant.

## Background

Growing old can lead to significant changes in most aspects of life, including physical, cognitive, and social well-being. For women in particular, ageing can be challenging due to menopause. Levine et al. conducted research on menopause accelerating biological ageing, concluding that the ageing process of cells speeds up by about 6% in women who have experienced menopause [1]. Additionally, a study that compared 4,116 women with menopausal symptoms to 4,695 women without, found that women who experienced menopausal symptoms had significantly more work impairment, healthcare utilisation, and lower health-related quality of life levels than those without symptoms [2].

Menopause indicates the termination of a woman’s menstrual cycle, impacting not just the reproductive system, with menopausal women more at risk of developing osteoporosis [3, 4]. Osteoporosis is a condition weakening the bones, making them fragile and easy to break, due to low bone mineral density and deterioration of bone tissue [5]. It develops gradually over the years and is diagnosed when a small impact causes a fracture of bones. Osteoporosis has been labelled the ‘silent thief’ as it is not painful; however, broken bones (e.g., in the spine) are a common cause of long-term pain [6–8]. Osteoporosis can be prevented and treated with bone-strengthening medicines and exercises. These include exercise, healthy dietary intake, Vitamin D supplements, and lifestyle changes like quitting smoking and reducing alcohol intake [9]. An understanding of the various factors that interact and affect the ageing process in a woman’s reproductive system will aid in developing strategies for handling menopause better and assist in understanding the process of biological ageing and the management of osteoporosis.

Advancements in technology in the form of mobile smartphones and applications (apps) have impacted our lives greatly. According to Degenhard [10], the number of smartphone users in the United Kingdom (UK) is expected to increase by 2.4 million users between 2022 and 2028. Laricchia [11] has also noted that smartphone usage has increased across all ages, but most noticeably among those aged between 55 and 64 years, increasing from 9% in 2012 to 90% in 2021.

The availability and accessibility of mobile phones have the potential to provide an efficient way to manage, track and understand menopause through mobile apps, consequently empowering women. A previous review of 22 menopause apps has found that numerous apps are available on the various operating systems that are designed to help manage menopause through education, tracking symptoms and risk assessments [12]. The review also found that involvement by medical professionals was only noted in 27.3% of the 22 menopause apps, and only 22.7% used evidence-based information [12]. This highlights important concerns about the quality of the apps overall and the reliability of the information being presented. However, the review did not use validated measures to assess the quality of the apps and did not focus on content related to osteoporosis.

This review aimed to explore and identify menopause apps available in the UK, assess their quality, functions, and content, and determine whether and to what extent they focus on menopause-related osteoporosis.

## Methods

A systematic review and content synthesis was conducted on the menopause apps available in the UK. Wherever relevant, this app review aligns with the updated Preferred Reporting Items for Systematic Reviews and Meta-Analysis (PRISMA) guidelines [13]. We searched the UK Apple App store and Google Play store for menopause apps available in the UK. The search was conducted between 14^th^ June and 30^th^ August 2022, and the keywords used to search for the apps were “menopause” and “menopausal”.

### Eligibility criteria

Menopause apps that were patient-focused or aimed at perimenopausal women were included. Apps were only included if they were in the English language, for individuals or groups and had a lifestyle focus (e.g., exercise, supplements).

Apps were excluded if they only looked at other aspects of women’s health, required external devices like wearables and virtual reality headsets, cost to download, or were symptom tracking/focussed apps (e.g., for hot flushes, mood changes, irregular periods, vaginal dryness, night sweats).

### App selection strategy

The apps were selected through a two-step process where one author (DP) screened the Apple and Google Play stores for menopause-related apps by reading the relevant app names and descriptions given. Duplicates of the apps were removed from both search platforms. The second step included downloading the apps and checking their eligibility by two independent authors (DP and NG) using the inclusion/exclusion criteria. Eligible apps were then individually reviewed by both authors. Any disagreements regarding eligibility were resolved through discussion.

### Data extraction items

Data extraction was informed by previous app reviews [14–16]. A Microsoft Excel coding sheet was created, and the two raters (DP and NG) extracted data from the apps. Table 1 describes the descriptive information of the apps reviewed. The descriptive information included the app name, version number, developer, markets available, cost, affiliation with a health body/university/charity and user rating – stars and the number of people rating it.

**Table 1:** Description of the data extraction items

**Data extracted**	**Description**
***Descriptive information***	
App name	Name of the mobile app
Version number	Version of the app reviewed
Developer	Name of developer
Market/s available	Google Play; Apple App
Cost	Free to download, in-app purchases
Affiliated with a professional (medical/health body or charity)	Yes/No
Average user rating	Not rated; the average number of public ratings (maximum 5 points)
Number of user ratings	Total number of user ratings
Total number of user ratings	Privacy policy, login, password, two-factor authentication
Third-party authorisations (e.g., data sharing)	Yes/No
Works offline	Yes/No
Works in the background	Yes/No
Asks to enable push notifications	Yes/No
***Content***	
Purpose	Educational; risk assessor; journal (symptom tracking)
Description	Summary of the app’s content
Content about Osteoporosis	Yes/No
Flesch Reading Ease	Scored 0 to 100
Flesch–Kincaid Grade Level	The score corresponds with the US education grade level
***Functionality***	
IMS Institute for Healthcare Informatics functionality score	Rated 1 (present) or 0 (absent) for the following functions: (1) inform, (2) instruct, (3) record, (3.1) collect data, (3.2) share data, (3.3) evaluate, (3.4) intervene, (4) display, (5) guide, (6) remind or alert, and (7) communicate
***Quality***	
Mobile App Rating Scale	19 items across four dimensions (engagement, functionality, aesthetics, and information quality) rated on a 5-point Likert scale: 1 = inadequate, 2 = poor, 3 = acceptable, 4 = good, and 5 = excellent

The content of the apps (see Table 1) was assessed by the primary focus of the app and its purpose (i.e., if it is educational, a risk assessor or for tracking). The app content also looked at whether the app focuses on osteoporosis or not and to what extent. Specifically, this determined whether the apps include the following information: risk calculation, education, lifestyle changes (e.g., exercise, supplements) or other related content.

The readability of the content was assessed using Flesch-Kincaid metrics (see Table 1). This was done by copying the content into a Microsoft Word document and using two Flesch–Kincaid metrics [17, 18]. The Flesch–Kincaid Reading Ease score ranges from 0 to 100, with higher scores indicating that the material is easier to read [17]. The Flesch–Kincaid Grade Level was also used, with scores referring to the equivalent grade level of education in the USA [18]. The technical content of the apps was determined by whether the apps contained a privacy strategy, mentioned third-party authorisations, and whether they asked to work in the background, work offline, or ask to enable push notifications.

Functions within the app were assessed based on the IMS Institute for Healthcare Informatics functionality score [19]. This scale has 7 items under 4 subcategories corresponding with specific functions. Items were rated 1 if the function is present and 0 if it is not. The total score was generated by summing the items and were ranging from 0 to 11. Scores between the raters were cross-compared.

The quality of the apps was assessed using the validated Mobile App Rating Scale (MARS) [20] as in other systematic app reviews [14–16, 21, 22], and the optional subscale for subjective quality was omitted to ensure that quality assessments were only objective. This scale contains 19 items across four dimensions (engagement, functionality, aesthetics, and information quality), with each item rated on a 5-point Likert scale. Mean scores from each rater were calculated for each MARS dimension and overall. A mean of the scores from each rater was then calculated and used for reporting the quality of each app.

### ORCHA approval ratings (if applicable)

All the apps were searched for in the Organization for the Review and Care of Health Apps (ORCHA) app library (https://appfinder.orcha.co.uk/). ORCHA is an organisation that objectively evaluates and reviews health and care apps. If they had been reviewed by ORCHA, the total overall score (%) for an app was noted, as well as scores for their three review domains (data privacy, professional assurance and usability/accessibility). Scores below 65% implied that the app had some issues, while a score below 45% highlighted the presence of considerable issues. According to ORCHA, these low-scoring apps can be considered unhelpful or unsafe.

#### Analysis

The analysis looked at the descriptive statistics and the inter-rater reliability scores for the MARS and IMS scales. The descriptive summary statistics consisted of a content synthesis of the apps reviewed. The inter-rater reliability statistics for the MARS and the IMS Institute for Healthcare Informatics functionality scores were done using IBM SPSS Statistics (version 27). An intraclass correlation coefficient using a 2-way mixed-effects, average-measures model with the absolute agreement was calculated for all MARS items, and a Cohens Kappa score was calculated for IMS.

## Results

**Fig. 1:**
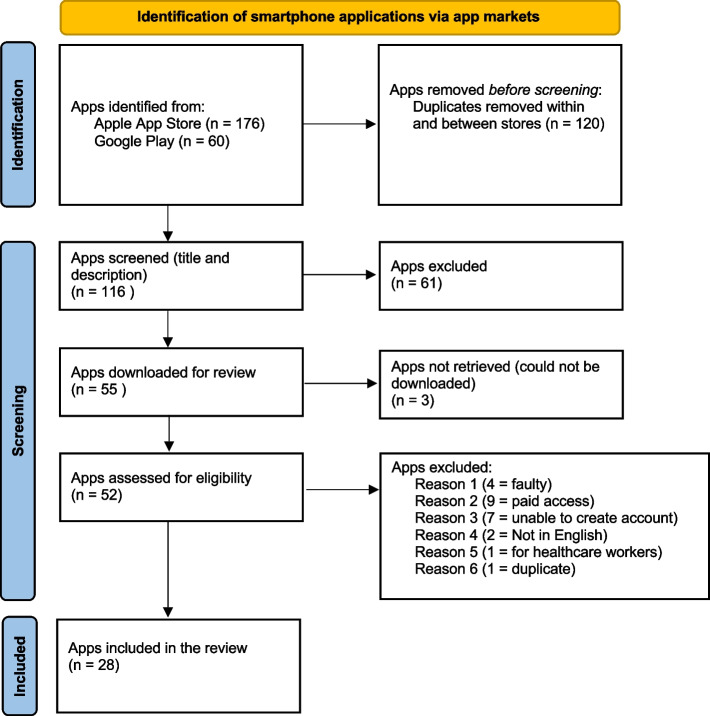
PRISMA flow diagram showing the app identification and screening process

The total number of menopause apps identified on the Apple and Google Play stores was 236. Before screening, 120 duplicates were removed within and between stores. A total of 116 apps were screened based on their title and description, during which 61 apps were excluded as they did not fit the criteria. Fifty-five apps were downloaded for review, out of which three apps were unretrievable (unable to be downloaded). A total of 52 apps were downloaded and assessed for eligibility. There were 24 apps excluded for the following reasons: four apps were faulty and would not open, nine required paid access, seven did not allow us to create an account, two apps were not in English, one app was for healthcare workers, and one was a duplicate with a different title. Eventually, a total of 28 apps were reviewed. Figure 1 presents the PRISMA flowchart summarising the search and screening process.

### Descriptive characteristics

Eleven apps were from the Apple Store (39%), and 10 (36%) were from the Google Play store. Seven apps (25%) were available in both stores. All apps were free to download, however; nine (32%) offered in-app purchases. Only one app (4%) was affiliated with a professional medical/health body or charity. The average user rating score was 4.48 out of 5 (range 0 to 5), and 14 (50%) apps had not been rated at the time of the review. The total number of user ratings was 17,400 (ranging from 3 to 11,914).

Of the 28 apps, two (7%) were able to work in the background, three (11%) worked offline, and 17 (61%) gave push notifications. Regarding privacy, 16 (57%) apps required a password and 18 (64%) required logins. Twenty (71%) had a privacy policy. Two (7%) apps required two-factor authentication, and nine (32%) indicated third-party authorisation (e.g., sharing data with an external company). Table 2 highlights some of the key characteristics of the apps reviewed.

**Table 2:** Key characteristics of the 28 menopause apps reviewed

**Characteristics**	**Number**	**Percentage**
***Privacy***		
Password	16	57
Login	18	64
Privacy policy	20	71
Two-factor authentication	2	7
Third-party authorisation	9	32
***Purchase costs***		
Free to download	28	100
In-app purchases	9	32
***Affiliation***	1	4
***App functioning***		
Works in background	2	7
Works offline	3	11
Sends push notifications	17	61

### Menopause and osteoporosis content

Out of the 28 apps, 18 (64%) aimed to educate the users, and 16 (87%) were journals, whereby the apps kept a record of menopause-related feelings and symptoms. Three of the apps (11%) were designed to calculate risk and three (11%) were designed for community interaction. One app shared menopause-related news (4%), and another one was designed to manage symptoms of menopause through hypnotherapy (4%).

More than half of the apps (*n* = 16; 57%) featured osteoporosis-related content. All of these were educational in their purpose. Their content usually included what osteoporosis is, its definition, the higher risk of osteoporosis during menopause, highlights on ways to prevent osteoporosis, lifestyle changes (e.g., diet, avoiding alcohol, sunlight exposure), the importance of supplements (e.g., calcium, Vitamin D), hormone replacement, building up bone strength and the importance of strength training. For example, the Megs Menopause app by the second screen ltd highlighted the pathophysiology of osteoporosis and suggested “Use it or lose it”– as muscles and bones need regular physical exercise to stay in their optimal shape.

Exercise programmes were recommended to prevent and manage osteoporosis. One of the apps focused on light exercises, including yoga, tai chi, and pilates and suggested a six-day exercise programme. Apps such as “Menopause guide” by King star studio and “Menopause: All information” by Android apps suggest specific types of exercise including high-intensity interval training and exercises using dumbbells and resistance bands. One app included a blog whereby osteoporosis was discussed by other users. Another app had links to the established FRAX [23] and QFracture [24] risk assessment tools, which are often used by nurses and doctors to calculate the risk of osteoporosis. One of the apps was also designed to track osteoporosis symptoms to help with early detection.

The readability of the apps was assessed using the Flesch-Kincaid metrics. On average, the Flesch Reading Ease score was 41.8 (range 2.8 to 87.1, SD: 17.33). The average Flesch-Kincaid Grade Level score was 13.09 (range 7.3 to 31.3, SD: 5.35) which corresponds to the US College graduate level. This means the content is difficult to read and best understood by university graduates.

**Fig. 2:**
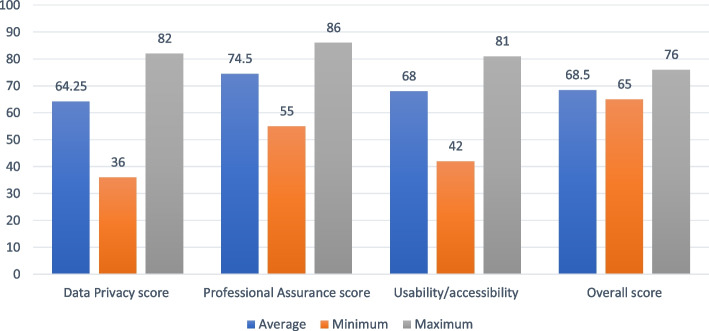
ORCHA scoring for the menopause apps reviewed

Four of the apps (14.29%) were reviewed by ORCHA. The average ORCHA score for data privacy was 64.25 (range 36 to 82, SD: 19.77), professional assurance was 74.5 (range 55 to 86, SD: 13.48) and that for usability/accessibility was 68 (range 42 to 81, SD: 17.61). The average overall score for the menopause apps reviewed by ORCHA was 68.5 (range 65 to 76, SD: 5.07). Figure 2 describes the average ORCHA scoring of the menopause apps reviewed.

### Functionality

The IMS Institute for Healthcare Informatics Functionality score was used to identify functions available within the apps. There was substantial agreement between the two raters’ scores of the apps reviewed, κ = 0.652 (95% CI, -0.568 to 0.736), *p* < 0.001.

**Fig. 3:**
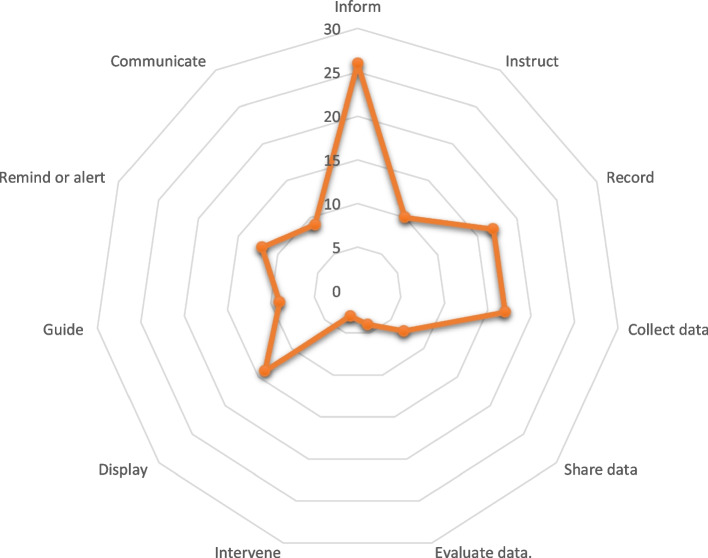
Radar graph showing the functions available in the reviewed menopause apps

The apps had an average function of 4.57 (SD: 3.01), ranging from one to nine functions. The radar graph in Fig. 3 highlights the most common function as inform (93%, *n* = 26), followed by record and collect data each (61%, *n* = 17), while the least common functions were to intervene (11%, *n* = 3) and evaluate data (14%, *n* = 4). The three apps namely Caria: Menopause & Midlife, Ask early Menopause, and Stella – Menopauserelief had nine functions, whereas nine of the apps reviewed had only one function which was to inform.

### Quality

The average overall quality for the apps reviewed was 3.1 (SD: 0.5) out of 5. Fourteen of the apps reviewed (50%) were below the minimum acceptability score of 3. Megs Menopause and Caria: Menopause & Midlife were the top-scoring apps that scored 4.1 and 4, respectively. On the other hand, the Ease Through Menopause app scored only 2.2 out of 5 and is the least scoring app. The average scores for the app quality were 2.98 (SD: 0.67) for engagement, 3.3 (SD: 0.5) for functionality, 3.1 (SD: 0.6) for aesthetics and 3.0 (SD: 0.6) for information. Most apps had good functionality scores ranging from 2.6 to 4.1. Engagement was scored the lowest comparatively, with scores ranging from 2.2 to 4.

It is to be highlighted that none of these apps was formally trialled or tested in peer-reviewed research. However, the information dimension also considers various other aspects like whether the app has specific and measurable goals, comes from a legitimate source, whether the visual information is clear and the quality and quantity of the content. The Caria: Menopause & Midlife app and the Ask Early Menopause app both scored the highest on this dimension (mean scores of 3.9). Similarly, the Balance—Menopause support app and the Health & her menopause apps both scored 3.8 for the dimension. Table 3 presents the reviewed apps with their quality scores for each dimension.

**Table 3:** MARS score under each dimension for the apps reviewed

App name	Developer	Version number	Mean engagement score /5	Mean functionality score /5	Mean aesthetics score /5	Mean information quality score /5	MARS overall mean score /5
Balance - Menopause support	Balance app limited	1.3.28	3.5	3.3	3.7	3.8	3.6
Health & her menopause app	Health and her ltd	1.2.0	3.8	3.8	3.7	3.8	3.8
Perry: Perimenopause community	pro.space	3.57.6	3.2	3.0	2.8	2.5	2.9
Healthy menopause	Cambridge digital Health	unavailable	2.8	3.5	3.0	3.2	3.1
Caria: Menopause & Midlife	Chorous Health Inc	1.9.12	4	3.9	4.0	3.9	4.0
Menopause	Sohila Zadran	5.0.2	2	2.9	2.3	2.2	2.4
Olivia	Menohealth AB	0.9.7	4	3.9	3.8	3.7	3.8
MiMa	Womaneze Inc	1.3.3	2.6	3.0	2.5	2.5	2.7
Stella - Menopauserelief	Vira Health Limited	1.4.1	3.9	3.5	3.7	3.2	3.6
VERGO: The Menopause Educator	Vergo Woman’s Health Network ltd	4	2.7	2.6	2.3	2.5	2.5
Ease Through Menopause	Dr. Jacob bargak	1.2	2.2	2.6	2.0	2.1	2.2
MenoBox - Menopause support	Lollipop Technology ( Hangzhou) Co., Ltd	1.2.0	2.8	3.4	3.7	2.7	3.1
Elda Health: Midlife & menopause Tracker	UPCALIBER technologies	1.0.15	3.3	3.1	3.5	3.4	3.3
EVIA: Hot flushes and Menopause	Mindset Health Pty Ltd	4.1	3.7	3.5	3.2	3.7	3.5
Responsum for Menopause	Responsum Health	1.6	3.5	2.6	2.5	2.8	2.9
MenoHealth	New Digital Marketing Limited	2.0.0	2.6	3.8	3.5	3.4	3.3
Mysysters Symptoms Tracker	Vorsdatter LLC	2.2.6	2.3	3.0	2.0	3.1	2.6
Ask early Menopause	Monash University	1	3.4	2.3	2.7	3.9	3.0
#1 Menopause Tracker	Usable Tech	1.0.5	2.9	3.6	3.5	3.0	2.8
Megs Menopause	Second Screen LTD	3.95	4.4	4.1	4.2	3.8	4.1
Menopause: All Information	Amdroid Apps	7.0	2.7	3.9	3.5	3.2	3.3
Menopause Guide	King Star Studio	2.7.68	2.4	3.8	3.0	2.8	3.0
Menopause tips	Free Apps For Everyone	1.1	2.2	3.4	3.0	2.6	2.8
Menopause early signs	H5akadimiy1	2.0	2.2	3.6	3.2	2.3	2.8
Guide to menopause	Digital Planete Space	1.1	2.3	3.6	2.7	2.4	2.7
Menopausal	M.A Company	1.0	2.7	3.4	2.5	2.8	2.8
Menopause Treatment	Everyone Learning Apps	1.3	2.5	3.3	2.2	2.6	2.6
Menopause (Details & Tips)	Amdroid Apps	1	2.7	3.3	2.7	2.8	2.8

### Cross-comparing the apps

The IMS and MARS scores were compared to identify the highest-scoring menopause app. The six apps which scored highest in either or both MARS and IMS are discussed in Table 4. The Apple App store contained most of the highest-scoring apps. The apps including Caria: Menopause & Midlife, Health & her menopause app, and Stella – Menopauserelief were able to track symptoms and suggested lifestyle changes to manage/prevent osteoporosis in menopause.

**Table 4:** Highest-scoring patient-facing apps considering the functionality and quality

App name	Marker	MARS^a^	IMS^b^	Description by the app developers
Caria: Menopause & Midlife	Apple store	4.0	9	Caria is designed to track and manage menopause symptoms, get personalized health insights, and connect with other women on a similar journey. Developed with health and wellness experts specializing in women’s health, Caria provides evidence-based approaches for managing menopause including cognitive behavioural therapy, hypnotherapy, nutritional therapy, mindfulness, and fitness.
Stella - Menopauserelief	Apple store	3.6	9	Stella’s cognitive behavioural therapy, pelvic floor physiotherapy, mindfulness and stress management techniques help make long-term lifestyle changes. Also, it helps to get support from human coaches, discover useful resources and join a supportive community. As per the description, it is noted that 75% of women who complete a Stella plan report improved symptoms. and 80% say they feel better.
Ask early Menopause	Apple store	3.0	9	This app helps to find trustworthy information of the highest quality from leading experts to learn about the condition and support with tools including a personal dashboard to help track symptoms, find the healthiest possible lifestyle and decide on the best management options.
Health & her menopause app	Apple store, Google Play store	3.8	8	This app empowers users through perimenopause and menopause by helping build positive lifestyle habits. The symptom toolkit includes cognitive behavioural therapy exercises for symptoms (e.g., hot flushes, night sweats and low mood), guided imagery meditation for better sleep, pelvic floor training and deep breathing for stress and anxiety.
Olivia	Apple store	3.8	7	Tailormade programs offer guidance on stress relief, better sleep quality, self-confidence and how to get better support from loved ones during this phase of life. These programs are created by experts and are based on cognitive behavioural therapy methods, based on the Greene Climacteric Scale.
Megs Menopause	Google Play store	4.1	6	MegsMenopause app is an everything-in-one menopause safe space app jam-packed with information. It says, “treat your menopause, don’t suffer in silence”.

^a^Overall mean score for the Mobile App Rating Scale (maximum score 5)

^b^IMS: overall score for the IMS Institute for Healthcare Informatics functionality score (range 0–11)

## Discussion

This app review has examined 28 patient-facing menopause apps available in the UK Apple App store and Google Play store. From the search process, it was evident that there are very few (*n* = 28) menopause-related patient-facing apps. Similar numbers were identified in the review by Gkrozou et al. in 2019, whereby 22 apps were reviewed out of 35 apps identified, and 91% of the apps were designed for consumers (patient-facing) [12].

Within the 28 apps we reviewed, only 57% (*n* = 16) had content related to osteoporosis. All the osteoporosis content was educational in purpose. The content usually included the definition and higher risk of osteoporosis during menopause, ways to prevent osteoporosis, lifestyle changes, the importance of supplements, hormone replacement, and the significance of exercise to reduce the risk of osteoporosis. However, this review only explored the presence (or absence) of osteoporosis content, rather than quantifying the content within the apps. Future work might focus on how comprehensive this content actually is.

Concerningly, none of the apps reviewed appeared to have been formally evaluated, assessing their acceptability and lifestyle changes like diet changes and exercise. Changing the approach to the development of authentic research-based healthcare apps involving the app developers, the users, experts in that field and researchers can make them more reliable and effective impacting the health and lifestyle of their users [25].

From the description of the apps, the development of the apps was not based on existing research evidence or peer-reviewed research, and only one was clearly affiliated with a medical/health organisation (Menopause app developed by Sohila Zadran affiliated with Igantia therapeutics). App development with an evidence-based approach is necessary to ensure that apps are usable, and effective in meeting their goals (e.g., behaviour change) and disseminate reliable and medically accurate information [25]. For instance, the app developed using a person-centred approach by Ryan et al. [26] has shown significant effects on behaviour change (i.e., lesser percentage of bone density lost). Ryan et al. [26] tested the efficacy of a theory-based, multifaceted, complex osteoporosis prevention smartphone app. The authors tested if the use of the app would improve bone mineral density and trabecular bone scores. They found that the implication of the person-centred osteoporosis app has a retention rate of 89.6% and the percentage of bone density lost was less than the national average. Alhussein and Hadjileontiadis [27] also concluded in their systematic review that most of the osteoporosis apps reviewed by them lacked clinically validated evidence of their efficacy and focussed on a limited number of symptoms. In addition, they emphasised the need for a more holistic and personalised approach within the app for the long-term self-management of osteoporosis. Further development of the content within osteoporosis apps is warranted through collaboration with medical experts. This is especially important in the UK as many healthcare apps are now regarded as medical devices, and emerging clinical apps may require approval from a regulatory body such as the UK Medicines and Healthcare products Regulatory Agency [28].

While the osteoporosis content was often limited in the apps, so was the inclusion of various functions. The average functionality score of the apps reviewed was 4.57 out of 11, which could be improved. Forbes Technology Council [29] have highlighted that a well-developed user-friendly app that allows users to easily accomplish what they wanted to do will keep them coming back to them. Additionally, they highlight that some features for successful and user-friendly mobile apps include targeted push notifications, personalisation, multi-device synchronization, multifactor authentication, and chat support. Future apps could also employ established behaviour change techniques, like personalization, prompts and cues, feedback and monitoring, and goals and planning [30]. These behaviour changes can also be mapped to Michie’s behaviour change taxonomy [31]. Sediva et al. [32] also highlight an opportunity for this in their systematic review on behaviour change techniques in digital health interventions for midlife women, indicating an overall weak use of theory, with an insufficient description of how specific behaviour change techniques were activated, low levels of treatment fidelity and insignificant outcomes.

The development of apps reviewed in this study did not appear to involve the end users. However, previous digital health interventions for women experiencing menopause reported in the literature involved end users during development and reported positive outcomes. Yeganeh et al. [33] co-designed digital resources with women with early menopause and health practitioners to address the information needs and support management. Their five-phase mixed methods multidisciplinary research used surveys, appraisal of clinical guidelines, digital resource development, evaluation and widespread dissemination of information, providing a model for successful interdisciplinary co-design research translation to improve women's health. Yeganeh et al. [34] later recruited 150 women to evaluate the co-designed menopause digital resource which included audio/video clips, a question prompt list, and information links. The authors found improved women’s health-related empowerment, illness perception, menopause symptoms, risk perception, and knowledge. In a participatory design project, Jakobsen et al. [35] developed mHealth approaches for women with osteoporosis by combining user-driven innovation and research. First, they identified user needs through qualitative work and then generated concepts through creative and mutual learning processes involving a team of women with osteoporosis, researchers, healthcare professionals and designers. The prototype app was developed and tested in an intervention involving healthcare professionals and women with osteoporosis in 2017. The study concluded that it was a useful tool to help women feel confident and reassured upon diagnosis of asymptomatic osteoporosis [36]. This previous work has highlighted that participatory and co-design approaches can be used to develop appropriate and effective apps which can evidence a significant desired outcome, reduced risk of osteoporosis. No such approaches were noted in the development of apps that were reviewed.

User testing by future end users (i.e., women experiencing menopause) and clinicians can also help to ensure that the content is reliable, and easy to read and understand, which is crucial to successful digital health technologies. In our review, the average reading age of the 28 apps was complex and best understood by university graduates. This is of concern as it may not match the general public’s literacy skills. The National Literacy Trust [37] highlighted the government survey of adult literacy skills and the Programme for the International Assessment of Adult Competencies (PIACC). The survey showed that in 2011, 14.9% of adults living in England (1 in 7) had literacy levels equivalent to the skills expected of a child aged nine to 11. It also indicated that 16.4% (or 1 in 6) of adults in England, and 17.4% (or 1 in 5) of adults in Northern Ireland, have literacy levels which are considered as ‘very poor literacy skills’ by the PIAAC. Additionally, one app in our review included the established fragility fracture FRAX and QFracture risk assessment tools [23, 24]. However, these are recommended for use by healthcare providers to predict the risk of hip fractures or osteoporotic fractures within 10 years and to inform treatment [23, 24, 38] and are not validated for self-assessment by users. Gilbert [39] stated that user-experience research should be the priority in developing user-centric products. This requires specialised researchers with a range of skills and experience in digital healthcare literacy, health psychology (including qualitative and quantitative methods), and experience in the clinical area in which the technology is being developed.

### Implications

From this review of menopause apps, it is evident that there are not many patient-facing menopause apps that are of high quality or have an acceptable ease of readability suitable to the literacy level of our target group. This highlights the need to design and develop high-quality menopause apps with an emphasis on osteoporosis. Experts in the field of menopause and the targeted users may be invited to contribute and update aspects of apps when relevant. They can also be involved in developing content that emphasises specific health problems during menopause, including osteoporosis. As discussed above, newer approaches like co-designed digital resources, participatory design and concepts like behaviour change strategies involving the target group and experts in the field may aid the development of innovative apps that are not just educational, but also able to track, monitor, interpret and give advice on aspects of osteoporosis during menopause. A similar conclusion was made by Senette et al. [40], who concluded that despite the explosion of health-related apps, no innovative examples are addressing a self-care approach to menopause by applying personalisation, adaptability, and persuasion to induce women to improve their health-related lifestyle.

It is also to be noted that the existing menopause apps in the UK market can be improved regarding their quality and functionality, with input from experts in that field and the targeted users themselves using techniques such as a user-centred design approach. For example, the usage of simpler language for patient-facing apps is required. Furthermore, the authentication of medical information shared on the apps and formal trials of the effectiveness of these apps is warranted.

### Strength and limitations

This review has some strengths and limitations that need to be addressed. Regarding limitations, only free apps related to menopause available at that particular period on the Apple Store and Google Play store were reviewed. This has the potential to change as there may be more apps available for use in the future, or some of them may no longer be available or have been updated. Also, only English language-based apps were reviewed. However, there are apps available in the market in other languages. Also, there may be apps available on the other operating systems, released privately, paid apps and apps with access restrictions which were not included. Nevertheless, there are some significant strengths for this app review including the use of Flesch–Kincaid metrics, the IMS and ORCHA scoring to provide measures of app readability and functionality. The MARS, a validated tool was also used to measure the quality of the apps.

## Conclusion

Prevention of osteoporosis is an important aspect of women’s health, especially during menopause, leading women to seek information and support on easily accessible smartphone apps. However, only half of the 28 apps we reviewed had content on osteoporosis, which was mostly educational in purpose. Few of these apps were acceptable regarding their functionality and quality. The readability of the apps needs to be simple for the general public to understand the content better. Additionally, collaboration with medical experts, user-centred design approaches [41, 42] and formal trials are required to ensure that content is appropriate and to test their usability and acceptability. This will help to ensure that there are reliable and good quality apps in the market to support healthy ageing.

## Data Availability

The list of the reviewed apps and their corresponding quality and functionality scores are available from the corresponding author on reasonable request.

## Abbreviations

PRISMA: Preferred Reporting Items for Systematic Reviews and Meta-Analysis
ORCHA: Organization for the Review and Care of Health Apps
MARS: Mobile App Rating Scale
SD: Standard deviation
FRAX: Fracture Risk Assessment Tool
UK: United Kingdom
USA: United States of America
